# RNA interference suppression of mucin 5AC (MUC5AC) reduces the adhesive and invasive capacity of human pancreatic cancer cells

**DOI:** 10.1186/1756-9966-29-53

**Published:** 2010-05-23

**Authors:** Sadaaki Yamazoe, Hiroaki Tanaka, Tetsuji Sawada, Ryosuke Amano, Nobuya Yamada, Masaichi Ohira, Kosei Hirakawa

**Affiliations:** 1 Department of Surgical Oncology, Osaka City University Graduate School of Medicine, Osaka, Japan

## Abstract

**Background:**

MUC5AC is a secretory mucin normally expressed in the surface muconous cells of stomach and bronchial tract. It has been known that MUC5AC *de novo *expression occurred in the invasive ductal carcinoma and pancreatic intraepithelial neoplasm with no detectable expression in normal pancreas, however, its function remains uncertain. Here, we report the impact of MUC5AC on the adhesive and invasive ability of pancreatic cancer cells.

**Methods:**

We used two MUC5AC expressing cell lines derived from human pancreatic cancer, SW1990 and BxPC3. Small-interfering (si) RNA directed against MUC5AC were used to assess the effects of MUC5AC on invasion and adhesion of pancreas cancer cells *in vitro *and *in vivo*. We compared parental cells (SW1990 and BxPC3) with MUC5AC suppressed cells by si RNA (si-SW1990 and si-BxPC3).

**Results:**

MUC5AC was found to express in more than 80% of pancreatic ductal carcinoma specimens. Next we observed that both of si-SW1990 and si-BxPC3 showed significantly lower adhesion and invasion to extracellular matrix components compared with parental cell lines. Expression of genes associated with adhesion and invasion including several integerins, matrix metalloproteinase (MMP) -3 and vascular endothelial growth factor (VEGF) were down-regulated in both MUC5AC suppressed cells. Furthermore, production of VEGF and phosphorylation of VEGFR-1 were significantly reduced by MUC5AC down regulation. Both of si-SW1990 and si-BxPC3 attenuated activation of Erk1/2. *In vivo*, si-SW1990 did not establish subcutaneous tumor in nude mice.

**Conclusions:**

Knockdown of MUC5AC reduced the ability of pancreatic cancer cells to adhesion and invasion, suggesting that MUC5AC might contribute to the invasive motility of pancreatic cancer cells by enhancing the expression of integrins, MMP-3, VEGF and activating Erk pathway.

## Background

Pancreatic cancer has a poor prognosis; the 5-year survival rate in only 3% and the median survival rate is only 6 months[1]. It is also associated with aggressive cancer cells, and metastatic disease that results from a lack of early-stage diagnostic methods and effective therapies. Adhesiveness and invasiveness of cancer cells play a central role in pancreatic cancer progression [2,3]. Mucins are highly glycosylated glycoproteins that are the major components of the viscous mucous gel covering the surface of epithelial tissues [4]. Changes in mucin expression or glycosylation accompany the development of cancer and influence cellular growth, differentiation, transformation, adhesion, invasion and immune surveillance [5]. Several papers have described the relationship between mucin and pancreatic cancer, for example, *de novo *expression of MUC5AC frequently occurs in intraductal papillary mucinous tumors and pancreatic adenocarcinoma [6-8], while Takikita et al. reported that borderline statistically significant associations are seen between expression of MUC5AC and shorter survival time in patients with pancreatic cancer [8]. However, the function of MUC5AC remains uncertain. In this study, we examined the impact of MUC5AC in a human pancreatic cancer cell line.

Small interfering RNA has recently been developed as a powerful tool to suppress the expression of specific gene products [9-11]. Previous studies on MUC1 suppression [10-12] in lung, breast and pancreatic cancer cells reported increased sensitivity to genotoxic drugs both *in vitro *and *in vivo *[11]. We down-regulated MUC5AC expression by siRNA and investigated the effects on the malignant and metastatic potential of human pancreatic cancer cell lines, SW1990 and BxPC3.

## Methods

### Cell lines and culture conditions

The human pancreatic cancer cell lines of SW1990, BxPC3 and PCI-64 were cultured in Dulbecco's modified Eagle's medium containing 10% fetal bovine serum, as described previously [13]. The stable cell line si-SW1990 and si-BxPC3, created by siRNA transfection of parental cells respectively, was maintained in the above medium containing 500 μg/ml Geneticin (Invitrogen Japan, Tokyo, JAPAN). Cells were cultured at 37°C under 5% CO2 in incubators with 100% humidity.

### Immunohistochemistry

Paraffin-embedded specimens from 100 patients with pancreatic ductal carcinoma who underwent resection at Department of Surgical Oncology, Osaka City University Hospital from 1995 to 2007 were stained with anti-MUC5AC monoclonal antibody (abcam, USA) according to the manufacture's protocol.

### siRNA design

The design of 19 nucleotide target sequences were based on a computer algorithm and 5'-GCCACCGCTGCGGCCTTCTTC-3' was selected as the target sequence. These were separated by a nine-nucleotide noncomplementary spacer (5'-TTCAAGAGA-3') from the reverse complement of the same 19-nucleotide sequence.

For preparation of recombinant plasmids, oligonucleotides (64 bp) were ligated into the mammalian expression vector, pSilencer 3.1-H1 neo (Applied Biosytems Japan, Tokyo, JAPAN) at the BamHI and HindIII cloning sites. Recombinant MUC5AC-pSUPER gfp-neo constructs were used to transform Escherichia coli DH5, which were selected on ampicillin-agarose plates and verified by sequencing.

### Cell proliferation assay

Cell proliferation was determined by the ^3^H-thymidine uptake assay. After 24 h or 48 h of incubation, radioactivity was measured using cell harvester and counters. Experiments were performed in triplicate, and values are expressed as cpm/well.

### Adhesion assay

The adhesion assay was done as described before [14]. Briefly, A 96-well microtiter plate was coated with Matrigel (2 μg/well), laminin (4 μg/well) and fibronectin (4 μg/well). Cancer cells (4 × 10^5^) were then seeded onto these components. No chemicals for extracellular stimulation were added. Cells were allowed to adhere to each well for 30 min at 37°C and were then gently washed three times with PBS. The adhesive cancer cells to extracellular components were evaluated by 3-(4, 5-dimethylthiazol-2-yl)-2, 5-diphenyl-tetrazolium bromide colorimetric assay (MTT assay). The percentage of cells adhering was calculated as follows: % binding = (absorbance of treated surface - ECM component)/absorbance of total surface × 100. All experiments were performed in triplicate.

### Invasion assay

Invasion activity of cancer cells was measured by the method of Albini *et al. *[15] with some modifications. Briefly, cancer cells (1 × 10^4^/ml, 200 μl) were seeded in the upper chamber separated with a 12-μm membrane filter coated with 50 μg of Matrigel without adding extracellular stimuli. After incubation for 72 h at 37°C, cancer cells invading the lower chamber were manually counted under a microscope. Six randomly selected fields were counted for each assay. Mean values from six fields were calculated as sample values. For each group, culture was performed in triplicate.

### RNA isolation and reverse transcription-polymerase chain reaction (RT-PCR)

Total RNA extraction and cDNA amplification were as described previously [16]. The following oligonucleotides were used for RT-PCR analysis: MUC5AC: 5'-TGCACCTGTGACAGCAGGAT-3' (sense), 5'-ACCTCCACCTTCCTATGGCT-3' (anti-sense); integrin α3: 5'-TACGTGCGAGGCAATGACCTA-3' (sense), 5'-TTTGGGGGTGCAGGATGAAGCT-3' (anti-sense); integrin α9: 5'-AGAGGAGGAGAGGGAACTGC-3' (sense), 5'-CCCAGACAGGTGGCTTGTAT-3' (anti-sense); integrin β3: 5'-GGGGACTGCCTGTGTGACTC-3' (sense), 5'-CTTTTCGGTCGTGGATGGTG-3' (anti-sense); MMP-3: 5'-GATATAAATGGCATTCAGTCCCTC-3' (sense), 5'-TCCTTGCTAGTAACTTCATATGCG-3' (anti-sense); and VEGF: 5'-CCTGGTGGACATCTTCCAGGAGTACC-3' (sense), 5'-GAAGCTCATCTCTCCTATGTGCTGGC-3'(anti-sense). The PCR condition as follows: predenaturation, 94°C for 10 min, denaturation, 94°C for 50 sec, annealing, 59°C for 50 sec; extention, 72°C for 1 min and final incubation, 72°C for 7 min. Other primers and PCR conditions were as described previously [16-19].

### *In vivo *experiments

For subcutaneous tumorigenicity, 1 × 10^7 ^cancer cells were injected into the flanks of BALB/c nude mice. For *in vivo *liver metastasis, 7.5 × 10^5 ^cancer cells were injected into the lower pole of the spleen under ether anesthesia. Mice were sacrificed after 5 weeks in order to measure the number of metastatic tumors in the liver. For *in vivo *peritoneal dissemination, 1 × 10^7 ^each cancer cells were injected into the peritoneal cavity, and the formation of peritoneal metastases was examined. Mice were sacrificed 14 days after injection, and peritoneal metastatic nodules were counted.

Animal studies were performed in accordance with the standard guidelines established by the Osaka City University Graduate School of Medicine. Six-week-old female Balb/c nude mice (Oriental Kobo, Tokyo, JAPAN) were used in all experiments, and five mice were used in each group.

### Measurement of VEGF in cell culture supernatants

For the generation of conditioned media, 1 × 10^5 ^cells were plated in a 6-well plate in growth medium and were allowed to attach overnight at 37°C. After washing with PBS, cells were moved to serum-free medium. After 24 h of incubation, conditioned medium was collected and VEGF concentrations were determined using a commercial human VEGF-specific enzyme-linked immunosorbent assay (R&D Systems, USA).

### Western blot analysis

Protein expression of VEGFR1, p-VEGFR1, MMP-3, Erk1/2, p-ERK and alpha3-integrin was examined by Western analysis. Cells grown to semiconfluence in 100-mm dishes were lysed in lysis buffer containing 20 mM Tris (pH 8.0), 137 mM EDTA, 100 mM NaF, 1 mM phenylmethylsulfonyl fluoride, 0.25 trypsin inhibitory units/ml aprotinin and 10 mg/ml leupeptin. Aliquots containing 50 μg of total protein were subjected to SDS-PAGE, and the protein bands were transferred to a polyvinylidene difluoride membrane (Amersham, Aylesbury, UK). Membranes were blocked with 5% nonfat milk or 5% FBS in Tris-buffered saline containing 0.1% Tween 20 at room temperature for 1 h and then incubated overnight at 4°C with mouse antihuman VEGF R1 antibody, rabbit anti-phospho-VEGF R1 antibody (R&D systems), mouse anti-MMP3 monoclonal antibody (MILLIPORE, USA), rabbit Erk1/2 polyclonal antibody, mouse p-ERK monoclonal antibody (SANTA CRUZ, USA), rabbit anti-human integrin alpha3 polyclonal antibody (MILLIPORE, USA) and beta-actin antibody (Cell Signaling, USA). After three washes, membranes were incubated for 1 h at room temperature with anti-mouse or rabbit IgG horseradish peroxidase-linked species-specific whole antibody (Amersham, USA). The three washes in TBST were repeated, and then the immunoreactive protein was detected using ImmunoStar Long Detection (WAKO, Tokyo, JAPAN).

### Statistical analysis

Student's t-test was used for statistical analysis. P values of less than 0.05 were considered to indicate statistical significance.

## Results

### Reduced expression of MUC5AC in SW1990 and BxPC3 cells

**Figure 1:**
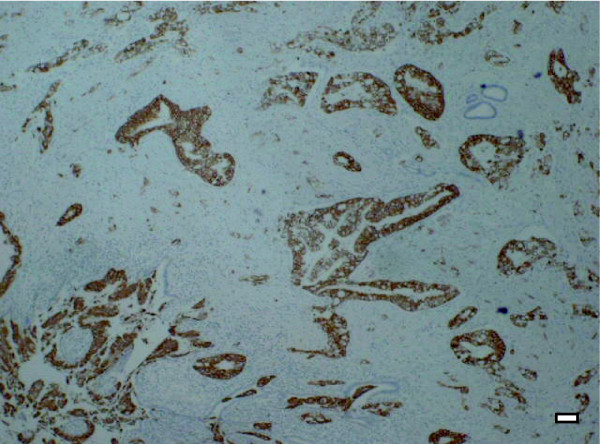
**Immunohistochemistry of MUC5AC**. Paraffiin-embedded tissues were stained using MUC5AC monoclonal antibody. Representative fileld of tumor tissue among 100 specimens of pancreatic ductal carcinoma showed MUC5AC protein expression (brown) limited to tumor epithelium. Scale bar, 50 μm.

**Figure 2:**
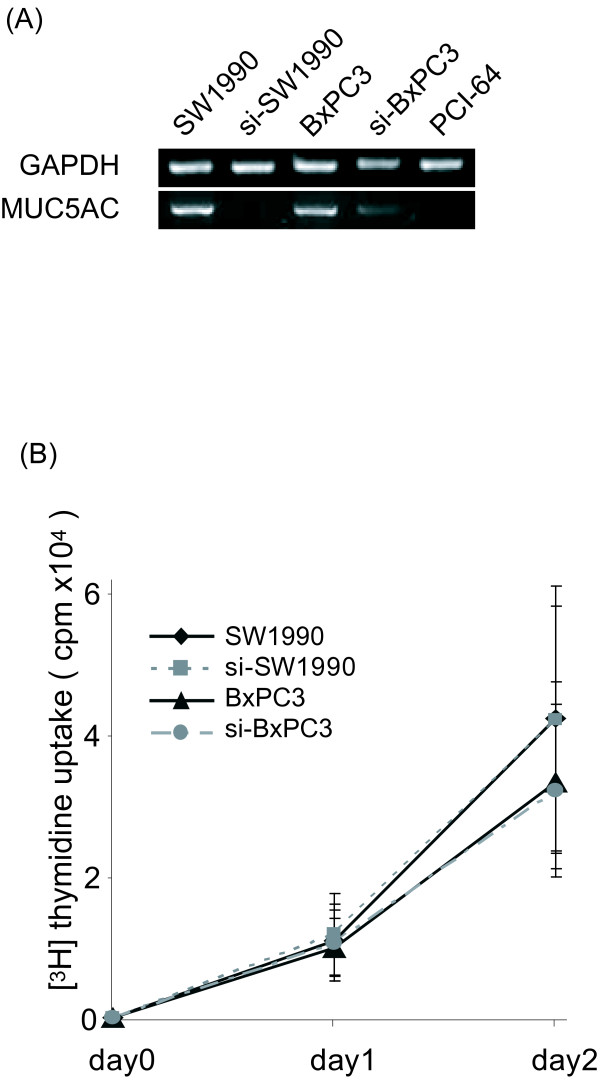
**Effect of si-RNA transfection on parental cells**. (A) Proliferation assay. Cell proliferation was measured by the [^3^H]thymidine uptake assay after 24 h or 48 h of incubation. Proliferation curve was plotted as radioactivity versus incubation time of cancer cells. No differences in proliferation were seen between si-SW1990 and p-SW1990. Shown data are means ± SD. (B) Detection of MUC5AC mRNA by RT-PCR. mRNA expression of MUC5AC decreased in si-SW1990 and si-BxPC3 compared with parental cells. PCI-64 has no MUC5AC endogeneously.

As Background, we tested MUC5AC expression in 100 specimens of pancreatic ductal carcinoma (Fig. 1). MUC5AC protein was detected in 85% of patients with pancreatic cancer, whereas no expression was observed in normal ductal tubular cells. Then, to examine the function of MUC5AC in pancreatic cancer cells, we delivered siRNA vector targeting MUC5AC into two human pancreatic cancer cells SW1990 and BxPC3 which were expressed MUC5AC. The resulting stable cell line, si-SW1990 and si-BxPC3, exhibited no expression of MUC5AC mRNA (Fig. 2A). As negative control, we confirmed no MUC5AC expression in PCI-64 cell (Fig. 2A). Also MUC5AC siRNA had no effect on the viability and form of SW1990 as well as BxPC3. The proliferative properties of transfectants did not differ from those of the parental cell lines (Fig. 2B). Doubling time of both cell lines were about 13 hours.

### Suppression of MUC5AC reduced the adhesive and invasive capacity of SW1990 and BxPC3 cells

**Figure 3:**
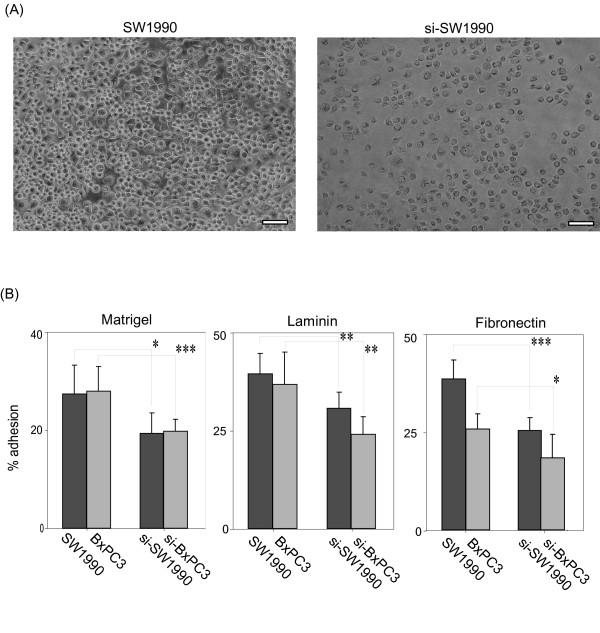
**Effect of MUC5AC suppression on cell adhesion**. (A) Cancer cells were seeded in 96-well plates coated with Matrigel, laminin and fibronectin. After 30 min incubation, adherent cells were quantified by MTT assay. A phase contrast photograph of SW-1990 shows the representative adhering cells to the well coated in finbonectin. Scale bar, 50 μm. (B) Quantitication of the effect of MUC5AC downregulation on cell adhesion to Matrigel, laminin and fibronectin. Cell adhesion of si-SW1990 and si-BxPC3 to ECM declined significantly compared with parental cells. Shown data are means ± SD. *; P < 0.05; **; P < 0.01; ***; P < 0.001.

**Figure 4:**
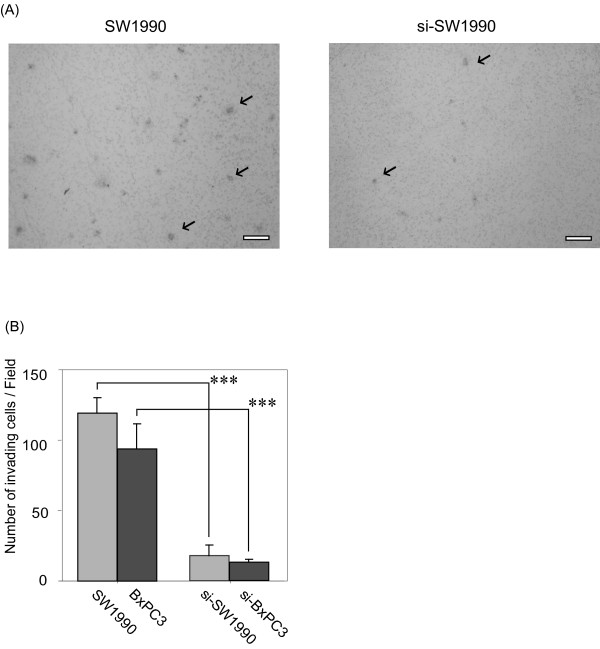
**Effect of MUC5AC suppression on cell invasion**. (A) Cell invasion through membrane filter coated with Matrigel was examined. 72 h later, invading cancer cells were stained by hematoxylin and counted under a microscope. A phase contrast photograph of SW-1990 shows the representative adhering cells to the well coated in finbonectin (arrows). Scale bar, 50 μm. (B) The number of invading si-SW1990 and si-BxPC3 was significantly lower compared to parental cells. Data shown are means ± SD. ***; P < 0.001.

Cancers grow through adhesion or invasion into interstitial tissue via extracellular matrix components (ECM). Then, we compared these properties between parental cell lines and siRNA transfectants (si-SW1990, si-BxPC3). We examined cellular adhesiveness to representative ECM of Matrigel, laminine and fibronectin, and evaluated cell viability si-SW1990 or si-BxPC3 adhering to ECM. The number of viable si-SW1990 was significantly reduced when compared with SW1990 (Fig. 3A). The percentage of adhesion to Matrigel, laminin and fibronectin decreased by 29% (P = 0.019), 22% (P = 0.008) and 34% (P = 0.0002), respectively (Fig. 3B). si-BxPC3 also revealed decrease of adhesion to three ECMs compared with BxPC3 (Fig. 3B). We then evaluated the invasive activity of cancer cells by Matrigel invasion assay. Through the 12-μm pore membrane, the number of migratory si-SW1990 cells significantly decreased by 85% compared with SW1990 (Fig. 4A, B). si-BxPC3 showed similar reduction of invasion to ECMs (Fig. 4B).

### Suppression of MUC5AC reduced expression of integrins and production of MMP-3 and VEGF

**Figure 5:**
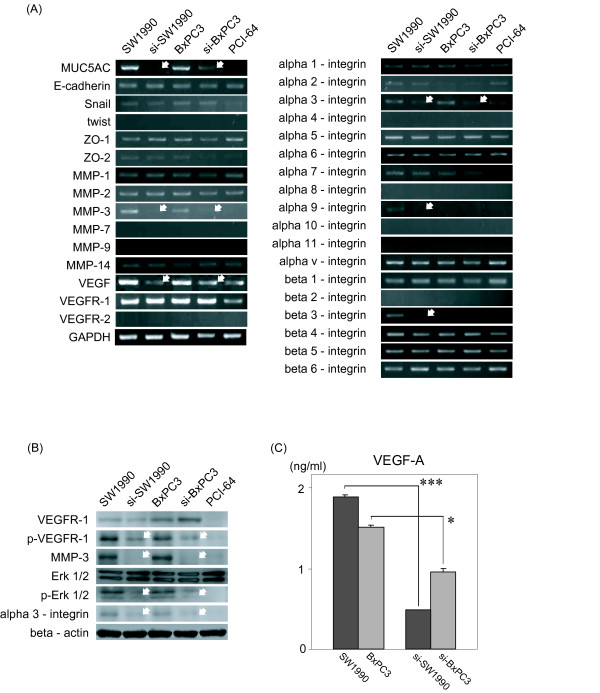
**mRNA and protein expression profiles of cancer cell lines**. (A) mRNA expression profile of cell lines. mRNA associated with cell adhesion and invasion was determined by RT-PCR. α3, α9 and β3 integrin, MMP-3 and VEGF mRNA expression (white arrows) of si-SW1990 was down-regulated, as compared to SW1990. MMP-3, VEGF, and α3-integrin were decreased in si-BxPC3. PCI-64 which expressed no MUC5AC did not reveal MMP-3, α3-integrin mRNA expression. (B) Western blotting. Reduced MMP-3 and alpha3-integrin expression in si-SW1990 and si-BxPC3 were verified by western blotting. Phosphorylation of VEGFR-1 and phosphorylation of Erk1/2 were decreased in both of si-SW1990 and si-BxPC3 compared with parental cells (white arrow). (C) ELISA. Cell culture supernatants were examined for production of VEGF-A. VEGF-A production by si-SW1990 and si-BxPC3 was significantly decreased than parental cells. Data are means ± SD. ***; P < 0.001.

In order to clarify the underlying mechanisms of these properties, we examined the mRNA expression of molecules associated with cell adhesion and invasion by RT-PCR. No differences were seen between SW1990 and si-SW1990 with regard to mRNA expression of E-Cadherin, Snail, ZO-1, ZO-2, MMPs and integrins, whereas mRNA expression levels of α3, α9, and β3 integrin, MMP-3 and VEGF had decreased in both of si-SW1990 as compared with SW1990. si-BxPC3 also exhibited lower mRNA expression of α3 integrin, MMP-3 and VEGF. No expression of VEGFR-2 and twist were detected (Fig. 5A). Next, we investigated production of MMP-3 and alpha 3-integrin proteins by cancer cells, resulting in higher expression level of these proteins by parental cells compared with MUC5AC suppressed cells (Fig. 5B). In addition, production of VEGF was significantly lower in the culture supernatant of si-SW1990 and si-BxPC3 (Fig. 5C). Having demonstrated that SW1990 and si-SW1990 cell express VEGFR-1 mRNA and produce VEGF, we finally examined phosphorylation of VEGFR-1 (p-VEGFR-1) and Erk1/2 on both cell lines by western blot analysis. Fig. 5B showed that VEGF induced VEGFR-1 phosphorylation were higher in both of SW1990 and BxPC3 compared with si-SW1990 and si-BxPC3. Moreover, Erk 1/2 phosphorylation was strongly reduced in MUC5AC reducing cells. On the other hand, PCI-64 which has originally expression of MUC5AC did not show mRNA expression of genes up-regulated in SW1990 and BxPC3 or the phosphorylation of VEGF-R or Erk1/2. No activation of Akt signaling was detected (data not shown).

### Effects of MUC5AC inhibition on si-SW1990 cell *in vivo*

**Figure 6:**
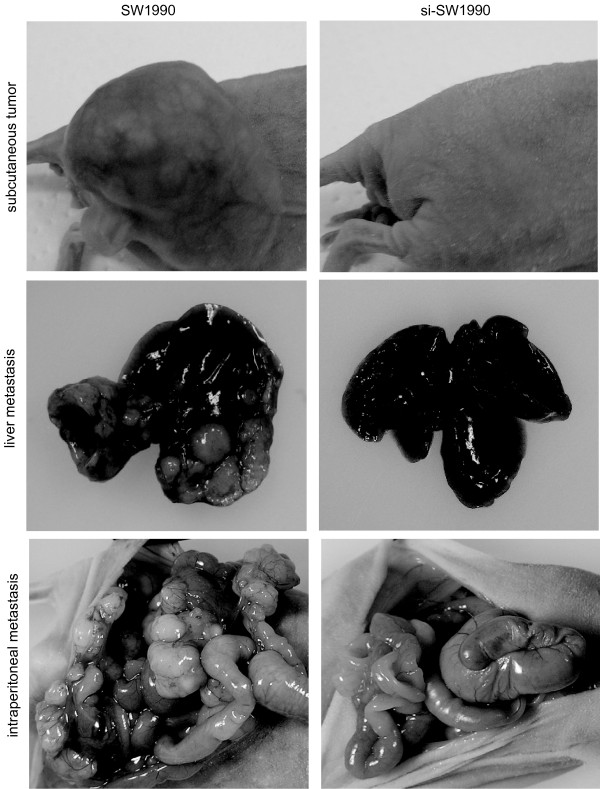
**Effects of MUC5AC suppression on SW1990 cell metastasis *in vivo***. Images of subcutaneous xenograft, liver metastasis and peritoneal metastasis following inoculation of SW1990 or si-SW1990. SW-1990 but not si-SW1990 formed a large subcutaneous nodule, and numerous nodules in the liver and intraperitoneal cavity.

In order to evaluate *in vivo *effects, we examined subcutaneous tumorigenicity, liver metastasis and peritoneal dissemination. Mice receiving inoculation of si-SW1990 cells showed no subcutaneous tumorigenesis (0%, 0/5), liver metastasis (0%, 0/5) or mesentery metastasis (0%, 0/5). In contrast, these metastases were seen in all mice inoculated with SW1990 (Fig. 6). As si-SW1990, inoculation of si-BxPC3 did not establish subcutaneous xenografts *in vivo*.

## Discussion

In this study, we have demonstrated that suppression of MUC5AC which was commonly expressed in pancreatic ductal carcinoma reduced adhesive, invasive and metastatic potential of pancreatic cancer cell lines. These results indicated that MUC5AC expression in cancer cells might be associated with invasive progression of pancreatic ductal carcinoma. It has been reported that mucins are associated with cancer growth. For example, MUC1 and MUC4 mucin augment cellular proliferation *in vivo *[12,20]. In our study, proliferation rate was not affected, although invasive and adhesive activities of SW1990 after MUC5AC inhibition were decreased markedly, suggesting that MUC5AC, similarly to MUC1 or MUC4, might have potential to accelerate progression of pancreatic cancer.

For cancer progression, several genes related to cell attachment, proteolysis and angiogenesis are important. We demonstrated that si-SW1990 reduced expression of α3, α9 and β3 integrins and MMP-3. Another MUC5AC down-regulated BxPC3 cells also decreased MMP-3, α3-integrin and VEGF. These results were supported by other reports. For example, Lohi reported that α3β1 integrin was a probable cell surface receptor acting with laminin-5 in the regulation of carcinoma cell invasion and proliferation [21]. α9β1integrin can mediate accelerated cell migration [22] and Hosotani demonstrated that α5β3 integrin expression is significantly correlated with lymph node metastasis and advanced stages of pancreatic cancer[23]. MMP-3 plays a crucial role in the insidious invasiveness of astrocytoma [24]. These results suggested that MUC5AC might augment malignant potential of pancreatic cancer cell such as MUC1 or MUC4. On the other hands, we found that PCI-64 cells by which MUC5AC was not originally expressed showed no augmentation of MMP-3, α3-integrin or VEGF, indicating that MUC5AC might not play a central role in progression of cancer like PCI-64 cells which have low level expression of MUC5AC.

Interestingly, we have observed significant decrease of VEGF-A production and VEGF-R1 phosphorylation by si-SW1990 and si-BxPC3 compared to parental cells. VEGF, a potent angiogenic mitogen, is linked to tumor growth, metastasis and poor prognosis for patients with pancreatic adenocarcinoma [25-28]. Association of VEGF with mucin has been reported. For example, immunohistochemistry of a combination of MUC1, VEGF and other two molecules was detected all ovarian cancer [29]. In non-small cell lung cancer, VEGF expression and MUC1 expression were independent prognostic variables [30]. Although we could not find reports about relationship of VEGF with MUC5AC, our results suggested that MUC5AC might have potential to regulate VEGF expression by cancer cells themselves.

Several studies have shown correlation among integrin, MMP and VEGF. An association between α5β3 integrin and MMP-2 activation was demonstrated in melanoma and breast cancer cells [23]. Expression of MMP-3 was induced by VEGF treatment in human endothelial cells. Recent studies have demonstrated that tumors and lymphangiogenic growth factors, such as VEGF-A and VEGF-C, induced lymphatic vessel expression of α4β1 integrin [31]. Our results showed that MUC5AC down regulation suppressed several integrins, MMP-3 and VEGF, indicating that down-regulated MUC5AC in pancreateic cancer might reduce production of VEGF-A resulting in suppression of integrins and MMP-3. However, our results did not demonstrate direct evidence that MMP-3 and α3 integrin suppressed by MUC5AC downregulation were associated with VEGF. Then we examined MAPK pathways in MUC5AC suppressed cells. Janes et al previously reported that pancreatic carcinoma cell lines expressed VEGFR-1, as well as VEGF and VEGFR-1 was capable of increasing MAPK signaling, migration, and invasion in an autocrine mechanism [32]. In this study, we have demonstrated that p-VEGFR-1 and p-Erk 1/2 of parental cells were down-regulated by MUC5AC suppressed cell lines. VEGF-A induced signaling cascade is mediated via activation of both of VEGFR-1 and VEGFR-2. We did not show phosphorylation of VEGF-R2 in this study, because SW1990 expressed no VEGFR-2 mRNA. Thus, our results suggest that MUC5AC positive pancreatic cancer cells might be activated the invasive potential via VEGFR-1 signaling pathway in an autocrine manner.

To clarify effect of MUC5AC on tumor, we tried to test it using mouse model *in vivo*, because our *in vitro *study has the limitation with regard to true tumor microenvironment. However, we found no subcutaneous tumorigenesis, intraperitoneal metastasis or hepatic metastasis after inoculation of MUC5AC suppressed cells. Several studies have reported that VEGF is believed to be essential for growth and metastasis of solid malignancies *in vivo *[27,33,34]. Fukusawa et al previously reported that pancreatic tumor growth and metastasis *in vivo *were significantly suppressed by a soluble VEGFR chimer which binds VEGF-A with high affinity [35]. Although we showed no direct evidence that MUC5AC was associated with tumorigenesis of pancreatic tumor, it was likely that inhibition of MUC5AC might reduce VEGF production by tumor *in vivo*. For future study, it should be necessary to investigate the mechanism for association of MUC5AC with tumorigenesis *in vivo*.

## Conclusions

The present work is the first demonstration of an association of MUC5AC with pancreatic cancer cell invasion. MUC5AC might contribute to the progression of pancreatic cancer by inducing adhesiveness and invasiveness in ECM via VEGF overexpression, indicating that MUC5AC may be a potentially target in the treatment of pancreatic cancer.

## Abbreviations

MUC5AC: mucin 5AC; siRNA: small interfering RNA; MMP: matrix metalloprotease; VEGF: vascular endothelial growth factor; MUC1: mucin 1; FBS: fetal bovine serum; MTT assay: 3-(4, 5-dimethylthiazol-2-yl)-2, 5-diphenyl-tetrazolium bromide (Sigma) colorimetric assay; RT-RCR: reverse transcription-polymerase chain reaction; ELISA: enzyme-linked immunosorbent assay; ECM: extracellular matrix components; MUC4: mucin4.

## Competing interests

The authors declare that they have no competing interests.

## Authors' contributions

SY carried out almost all studies and performed the manuscript. HT and TS supported with design and interpretation of this study. Statistical analysis was carried out by SY and RA. NY provided and participated in ELISA. Overall supervision of the manuscript was completed by KH. Financial correction was performed by HT and KH. All authors read and approved the final manuscript.
